# Development of a screening program to assess motor function in the adult population: a cross-sectional observational study

**DOI:** 10.1007/s00776-015-0737-1

**Published:** 2015-05-26

**Authors:** Toru Ogata, Shingo Muranaga, Hideaki Ishibashi, Takashi Ohe, Ryoichi Izumida, Noriko Yoshimura, Tsutomu Iwaya, Kozo Nakamura

**Affiliations:** Research Institute, National Rehabilitation Center for Persons with Disabilities, 4-1, Namiki, Tokorozawa, Saitama Japan; Kameda Medical Center, Chiba, Japan; Department of Orthopaedic Surgery, Ina Hospital, Saitama, Japan; Department of Orthopaedic Surgery, NTT Medical Center Tokyo, Tokyo, Japan; Keiyu Joint Reconstruction Center, Edogawa Hospital, Tokyo, Japan; Department of Joint Disease Research, 22nd Century Medical and Research Center, Faculty of Medicine, University of Tokyo, Tokyo, Japan; National Rehabilitation Center for Persons with Disabilities, Saitama Tokorozawa, Japan

## Abstract

**Background:**

Motor dysfunction is a major reason why the elderly lose their independence in their daily lives. The concept of locomotive syndrome has been proposed to describe the risk of mobility dependence caused by various locomotive organ disorders. The preservation of locomotive organs is now socially important in the middle-aged and geriatric population. Therefore, it is important to establish a screening program to evaluate motor function and related quality of life in a wide range of ages.

**Methods:**

We propose a new set of pre-existing scales (the Two-Step test, Stand-Up test, and 25-question Geriatric Locomotive Function Scale) as screening tools to identify the population at high risk for locomotive syndrome. We performed a preliminary survey on 777 subjects who had no apparent disorders related to motor function. We also examined the reliability of the Two-Step test and Stand-Up test.

**Results:**

We found that each scale did not show ceiling or floor effects in various age groups. Because the correlations between the three scales were significant but weak, we assume that each scale covers different aspects of mobility. The test–retest reliability was found to be satisfactory for the Two-Step test and the Stand-Up test.

**Conclusion:**

Our results suggest that our “Short Test Battery for Locomotive syndrome” is a feasible and reliable tool for screening the adult population as a preventative strategy for locomotive syndrome in a super-aged society.

## Introduction

Motor function is one of the components associated with quality of life, especially in the geriatric population. Since the level of mobility is directly associated with activities of daily living (ADLs), various attempts have been made to develop optimal treatments for bone and joint disorders such as osteoarthritis, hip fracture, and lumbar canal stenosis [1]. “Locomotive syndrome” is a clinical entity which describes mobility dysfunction originated from pathologies in locomotive elements such as bone, cartilage, muscle, and the nervous system [2, 3]. The concept suggests the importance of allocating the condition of each motor element in the overall mobility function. From the view point of public health, an effective screening tool is necessary to identify the high-risk group, for which either preventive intervention or medical treatment would be required [4, 5]. Such screening tool is also important to promote consciousness about mobility in the general population, so that people can understand whether they should alter their lifestyle based on their physical activities [6].

Seichi et al. [7] developed a self-answering questionnaire, called the “25-question Geriatric Locomotive Function Scale (GLFS-25)” to evaluate motor dysfunction in the aged population (older than 65 years). They reported the feasibility of the score by showing that the GLFS-25 score correlates with objective mobility rated by medical experts. Together with other physical assessment tools, the GLFS-25 is expected to be used for screening to identify the population at high risk of losing their independence in locomotion. Regarding functional assessment, a recent cohort study has proved that slow walking speed and an increased time required to stand up from a chair without using the arms (Chair-Stand test) are among the risk factors for the need of nursing care [8]. Therefore, functional evaluations for those indicators would be good screening tools together with the GLFS-25.

From the view point of preventive medicine, screening systems are also required to identify not only the geriatric population at risk, but also younger generations. A proper classification of the younger population according to their mobility would be beneficial for implementing a guideline for exercise habit to reduce the incidence of mobility-related problems in the whole population. However, the selection of assessment tools for mobility in the population aged between 30 and 70 years is not easy, because the problems of “floor effect” or “ceiling effect” make it difficult to obtain proper data.

Herein, we propose a new screening program for the population aged between approximately 40 and 70 years, to assess the mobility and potential risk for future dysfunction. Our program consists of two functional tests (the Two-Step test and Stand-Up test) and the GLFS-25. As a pilot study, we surveyed 777 adults and confirmed the feasibility of the program.

## Materials and methods

### Subjects

This cross-sectional pilot study was conducted at the work place, health promotion lecture meetings, and periodic medical check-ups. A sample of 777 adults was recruited for the screening test. Inclusion criteria were as follows: aged 20–90 years without specific mobility disorders. The recruitment was adjusted so that the subjects were evenly distributed according to age group and sex. The subjects provided written informed consent prior to participating in the study, which was approved by the ethical committee at the National Rehabilitation Center for Persons with Disabilities.

### Functional tests

**Fig. 1:**
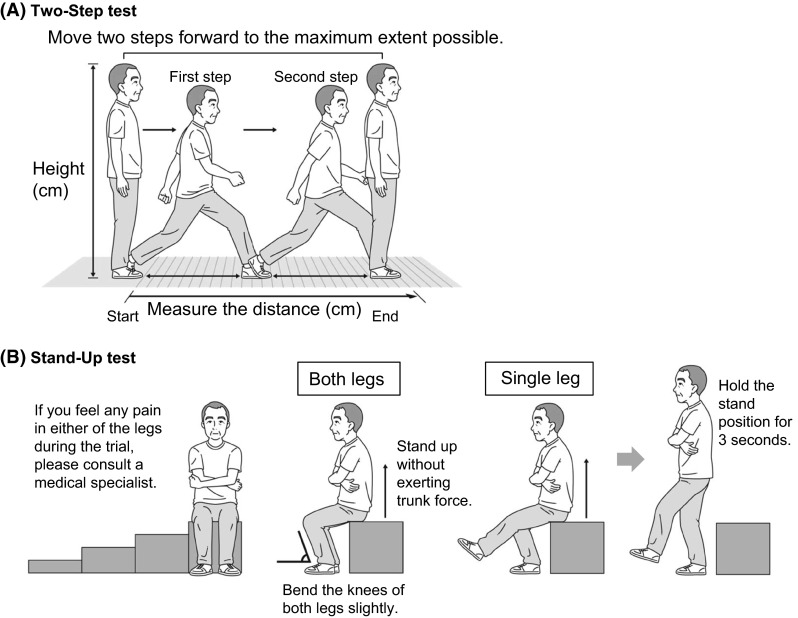
The schematic procedure of the Two-Step test (**a**), and Stand-Up test (**b**)

For functional tests, we adopted the Two-Step test and Stand-Up test. The Two-Step test, shown in Fig. 1a, which was previously examined by Muranaga et al. [9] has been developed as a screening tool for walking ability. The subject starts from the standing posture and moves two steps forward with maximum stride with the caution not to lose balance. If the subject succeeds in holding the final standing position longer than 3 s without any additional steps, the trial is judged as completed. The distance is then standardized by dividing it by the subject’s height. The test is performed twice, and the best result is recorded. Muranaga et al. [9]. reported that the value of the Two-Step test has a strong correlation with maximum walking speed. The Stand-Up test, shown in Fig. 1b, was also developed by Muranaga et al. [10] and is performed with stools of 10, 20, 30, and 40 cm in height. Subjects are requested to stand from each stool with one leg or two legs. If the subject succeeds in holding the final standing position longer than 3 s without any additional steps, the trial is judged as completed. A 0–8 score is allocated to the performance as shown in Table 1. Muranaga et al. [10] reported a significant correlation between the Stand-Up test score and the weight bearing index which is calculated as knee extension torque divided by body weight. To evaluate the reliability of these functional tests, we examined test–retest reproducibility. For that purpose, another 88 subjects were recruited and performed the Two-Step test and Stand-Up test two times each with 5–9 day intervals.

**Table 1:** Scoring system of Stand-Up test

	Two-leg stand					One-leg stand			
Height	Fail at 40 cm	40 cm	30 cm	20 cm	10 cm	40 cm	30 cm	20 cm	10 cm
Score	0	1	2	3	4	5	6	7	8

One-leg stand requires subjects to succeed at indicated height in both *right* and *left* leg

### Questionnaire

The subjects were asked to fill out the GFLS-25 questionnaire, which consists of 25 items with a score of 0–4 for each item. The total score (ranging from 0 to 100) was used for analyses, in which the higher score indicates a worse condition.

### Statistical analysis

SPSS version 17 software (SPSS, Chicago, IL, USA) was used for all statistical analyses. Correlations between the scales were examined by Spearman’s rho test. A *p* value <0.05 was considered to indicate statistical significance. The interaction between sex and age in the functional test were examined by using either Two-way analysis of variance (ANOVA) or the Mann–Whitney U test followed by Bonferroni correction. For test–retest reliability, the intraclass correlation coefficient (ICC) was calculated for the Two-Step test, and the Kappa value was calculated for the Stand-Up test.

## Results

### Characteristics of the subjects

**Fig. 2:**
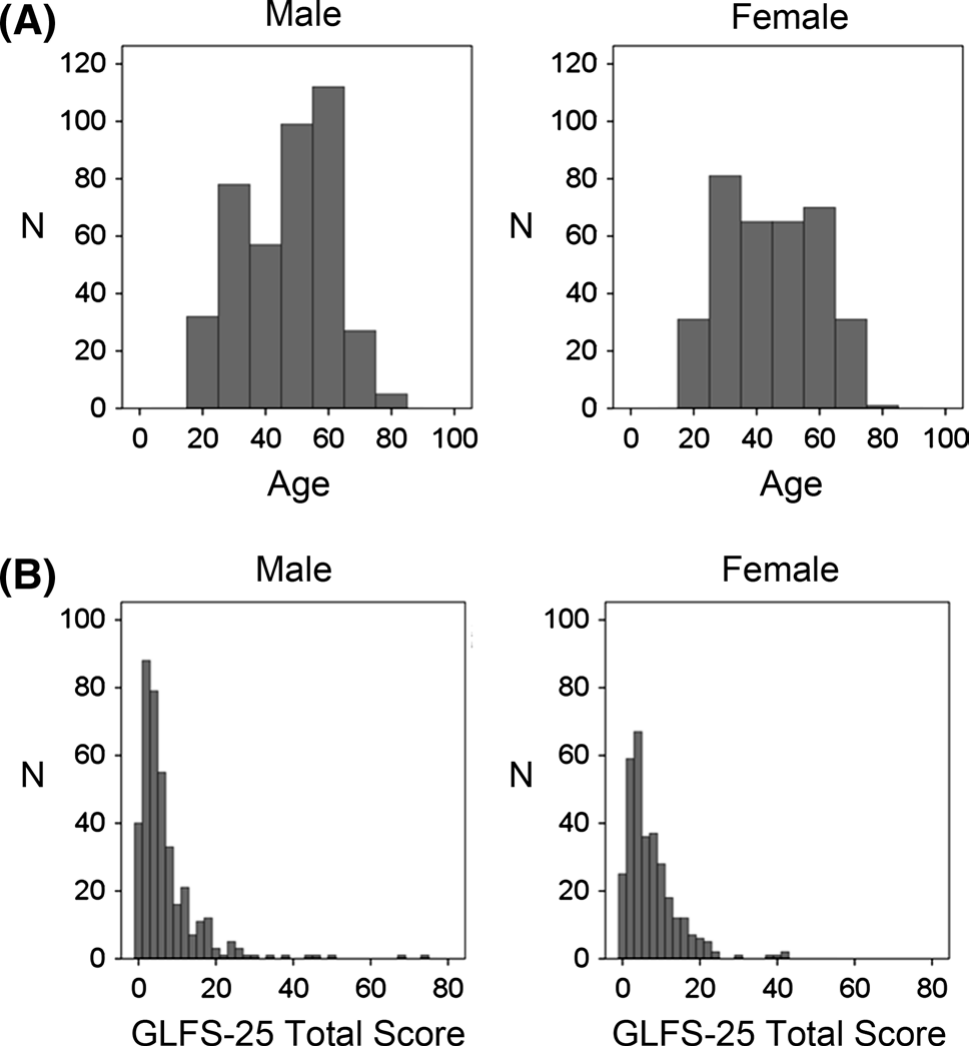
**a** Age distribution of the subjects. **b** Prevalence of the 25-question Geriatric Locomotive Function Scale (GLFS-25) total score

In the current study, we recruited subjects from work places, medical check-ups, and health-related seminars. Table 2 and Fig. 2a show the demographic background and the age distribution of the subjects, respectively. The majority of the subjects did not appear to have bone, joint, or muscle disorders; this was reflected in the mean GLFS-25 score (men: median, 4 [95 % confidence interval (CI), 4.0–5.0]; women: median, 5 [95 % CI, 4.0–6.0]) (Fig. 2b). About 10 % of the subjects (men, 10.2 % and women, 9.7 %) scored ≥16 in the GLFS-25 questionnaire, which has been reported to be the cut-off point to discriminate people with motor dysfunction symptoms from healthy people.

**Table 2:** The demographic background of the subjects

	Male (*N* = 421)	Female (*N* = 356)
Age	46.9 (22–85)	44.6 (21–80)
Height (cm)	169.4 (137.7–191.0)	156.3 (143.0–173.0)
Weight (kg)	68.0 (41.3–107.0)	52.8 (34.1–91.8)
Body Mass Index	23.7 (14.9–39.1)	21.6 (14.3–38.7)
Ratio of GLFS-25 ≥ 16 (%)	10.2	9.7

### Reliability of the Two-Step test and Stand-Up test

**Fig. 3:**
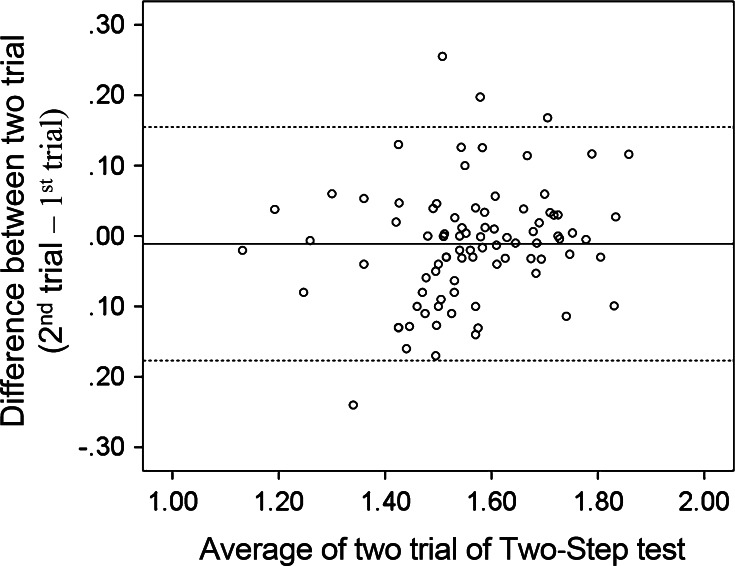
The Bland and Altman plot displays the agreement of the two trials of the Two-Step test with 5–9 day intervals. The *line in the graph* indicates the mean of the difference between the first and second trial. The *dashed line* indicates mean ± 2SDs

As the Two-Step test and Stand-Up test are relatively new functional tests, we examined the test–retest reliability of these tests with 5–9 day intervals. The demographic data of the 88 subjects (men 30; women 58) are shown in Table 3. The ICC for the Two-Step test was 0.84 (95 % CI 0.77–0.89). Furthermore, Bland and Altman analysis revealed that there was no bias between the first and second measurements (mean, −0.011; 95 % CI −0.0283 to 0.0070) (Fig. 3). As for the Stand-Up test, the non-parametric measurement, the Kappa coefficient was 0.731 (*p* < 0.0001), representing the agreement between the two measurements.

**Table 3:** The demographic background of the subjects for test–retest analysis

	Male (*N* = 30)	Female (*N* = 58)
Age	48.2 (23–75)	52.4 (19–88)
Two-Step test	1.62 (1.38–1.82)	1.54 (1.14–1.88)
Stand-Up test	5 (3–8)	5 (1–8)

Mean (range) for age and Two-Step test; median (range) for Stand-Up test

### Age-dependent elevation of the GLFS-25

**Fig. 4:**
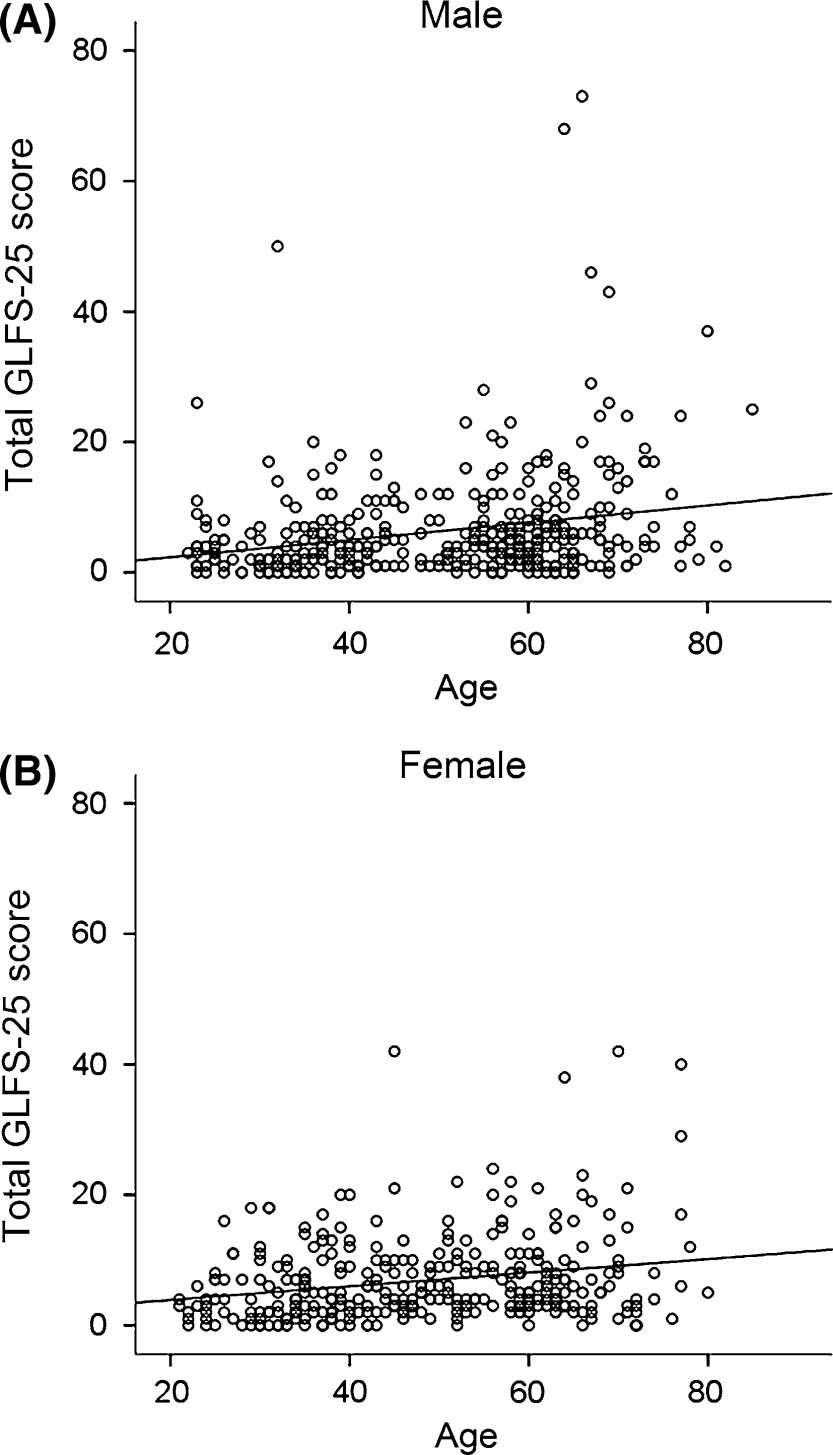
Scatter plotting of the 25-question Geriatric Locomotive Function Scale (GLFS-25) total score and age of each subject among men (**a**), and women (**b**). The line is drawn as a regression line between two parameters

We utilized the GLFS-25 as a tool to evaluate subjective health in motor functions. As shown in Fig. 4, the distribution of the score shows a gradual increase in accordance with age in both men and women. Statistical analyses revealed a correlation between the GLFS-25 total score and age in both men (*r* = 0.258, *p* < 0.001) and women (*r* = 0.210, *p* < 0.001) (Fig. 2a, b). The proportion of men and women who marked the lowest score (“0” in GLFS) was 10.4 and 7.8 %, respectively.

### Age-dependent decline in the Two-Step test

**Fig. 5:**
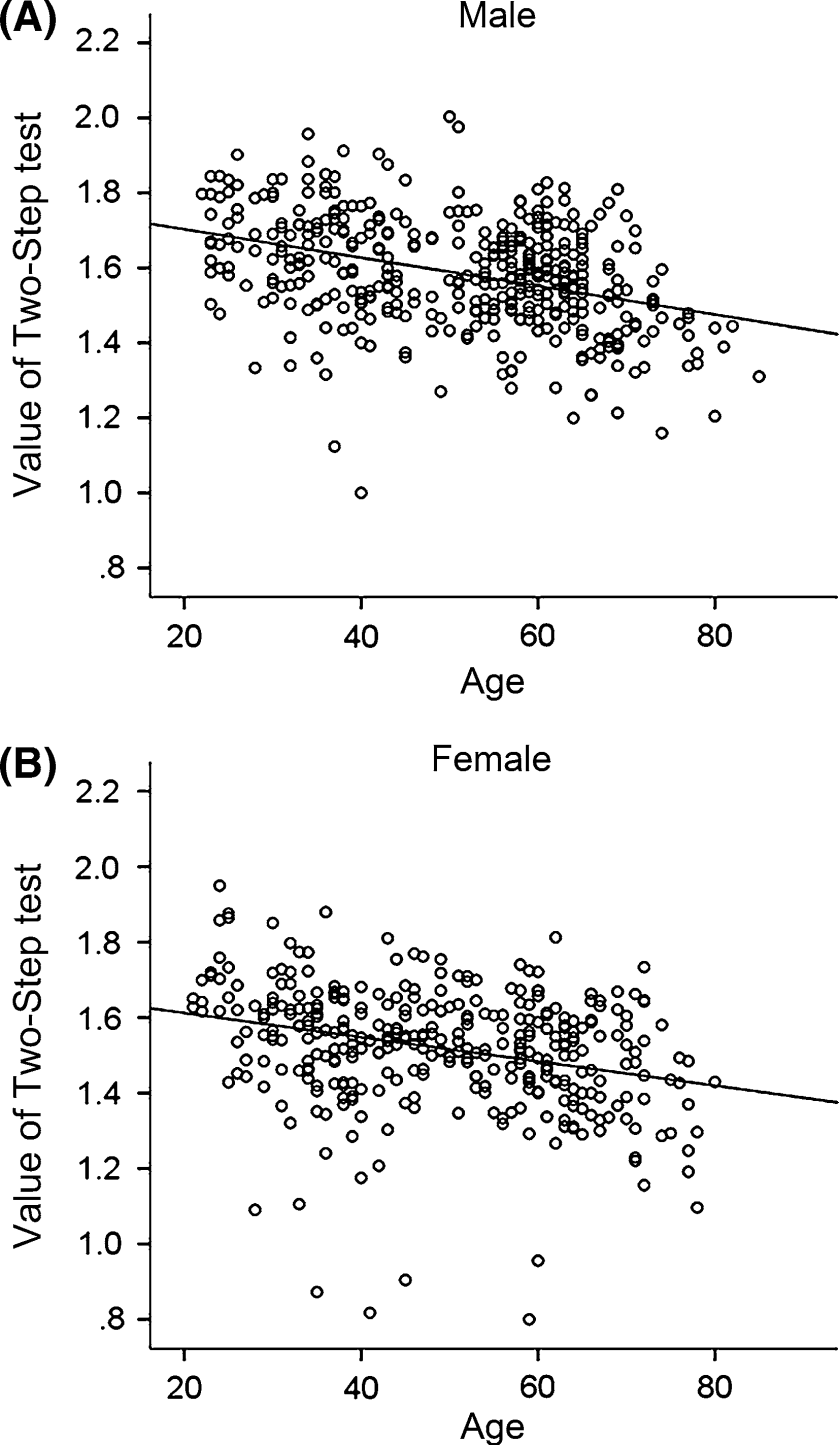
Scatter plotting of the Two-Step test and age of each subject among men (**a**), and women (**b**). The line is drawn as a regression line between two parameters

We performed the Two-Step test, which is well correlated with walking speed. There were differences according to age. Figure 5 shows the negative correlation between the Two-Step test index and age in both men (*r* = −0.375, *p* < 0.001) and women (*r* = −0.321, *p* < 0.001) (Fig. 5a, b). We observed no ceiling effect in this functional test. The two-way ANOVA test revealed that there was no interaction between sex and age (categorized into 10-year age groups) (*F*[5, 742] = 0.767, *p* = 0.573).

### Age-dependent decline in the Stand-Up test

**Fig. 6:**
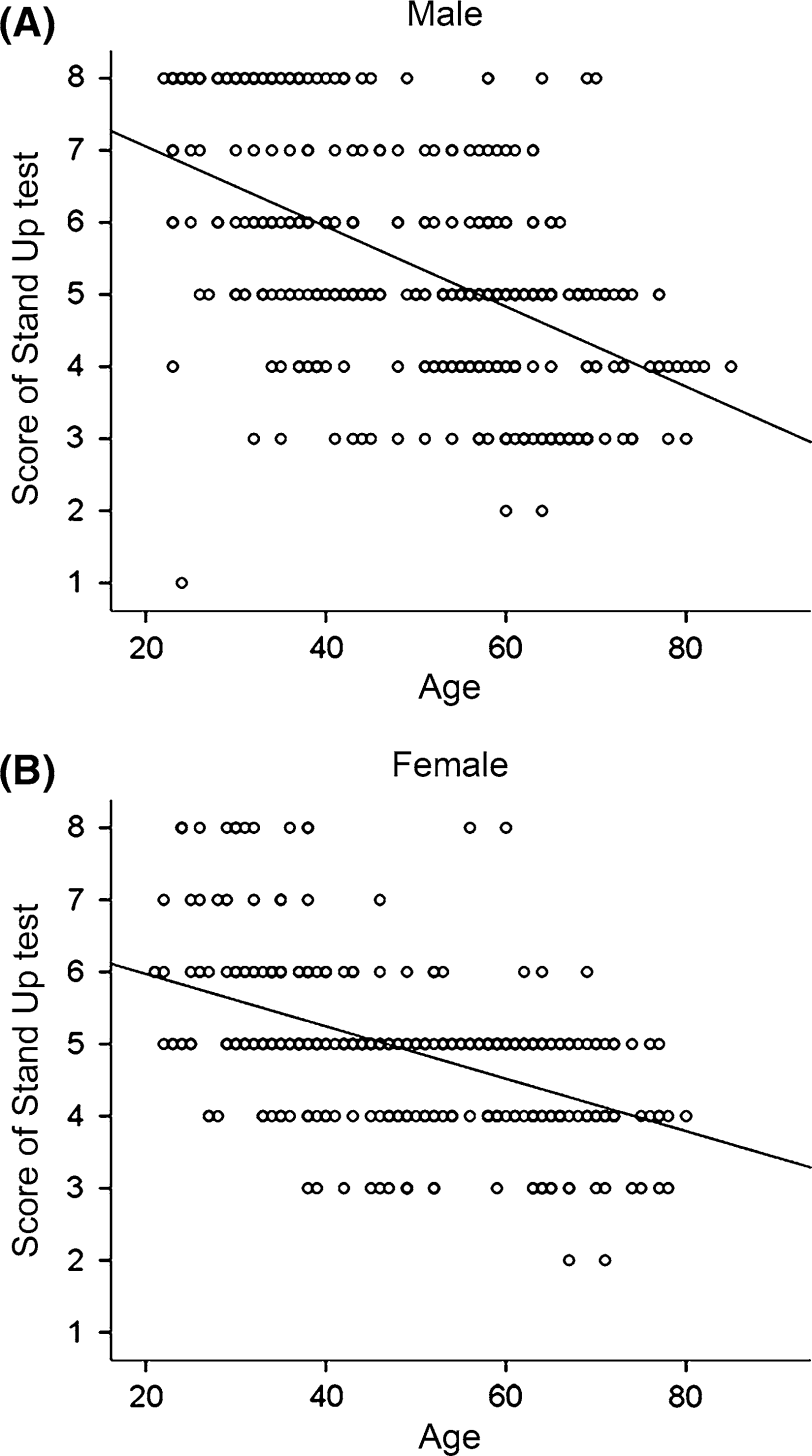
Scatter plotting of the Stand-up test and age of each subject among men (**a**), and women (**b**). The line is drawn as a regression line between two parameters

The Stand-Up test score is a non-parametric value, in which higher scores indicate better performance. Similar to the Two-Step test, we observed a negative correlation between age and the Stand-Up test score (men: *r* = −0.522, *p* < 0.0001; women: *r* = −0.506, *p* < 0.0001) (Fig. 6a, b). The maximum score, which is 8, was marked by 14.1 % of men and 4.1 % of women. As for the difference between sex and age (categorized into 10-year age groups), the score among men was higher than that of women in their 20 s, 30 s, and 40 s (Mann–Whitney *U* test, *p* < 0.001), while the differences were not significant for subjects in their 50 s, 60 s, and 70 s (Mann–Whitney *U* test, *p* = 0.107, 0.44, and 0.302, respectively).

### Correlation between each scale

**Fig. 7:**
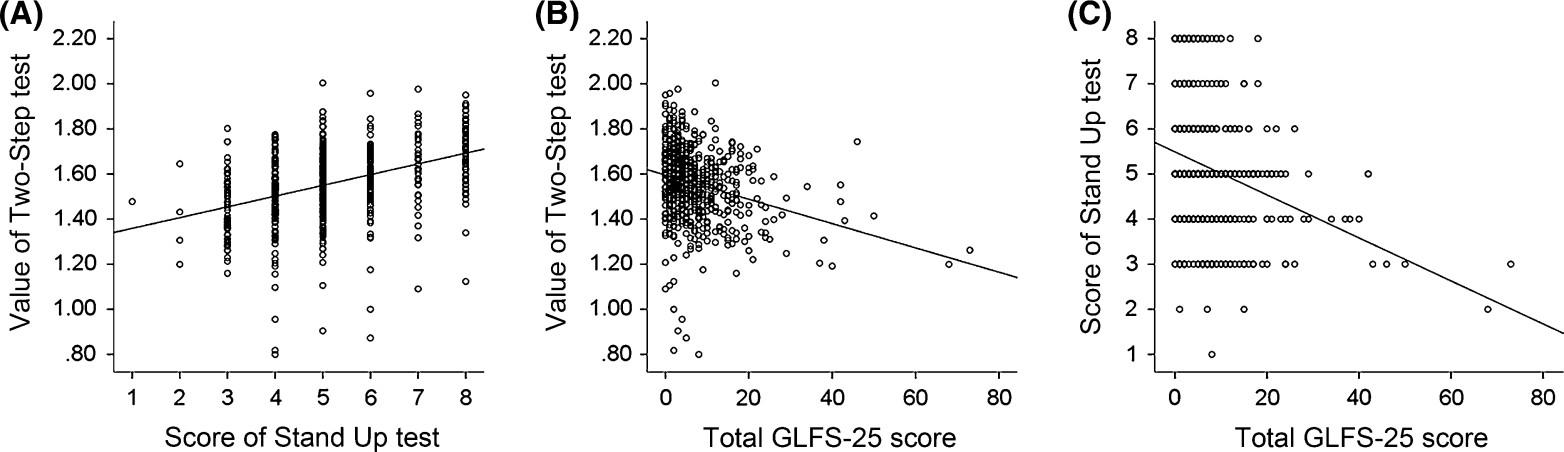
Scatter plotting of Two-Step test and Stand-Up test (**a**), Two-Step test and GLFS-25 (**b**), and Stand-Up test and GLFS-25 (**c**). Each plot includes both men and women. The line is drawn as a regression line between two parameters

Both the Two-Step test and the Stand-Up test are related to lower limb function, and we examined the correlation between the two scales. Because both parameters are correlated with age, we examined the partial correlation between them. We found a week correlation between the Two-Step test and the Stand-up test (partial correlation, *r* = 0.308, *p* < 0.001) (Fig. 7).

## Discussion

In the present study, we utilized two functional tests and one self-questionnaire to assess the motor function in the adult population including middle-aged and elderly subjects. While performing the survey, we did not observe any accidental episode such as falling.

The independence of daily living is affected by many factors, including internal dysfunction, motor dysfunction, and mental dysfunction. Since motor dysfunction is one of the top five leading causes of certified need of care, it would be reasonable to focus on motor function for screening and intervention, to improve the level of independence in the geriatric population [11–13]. For evaluating geriatric motor function, the Short Physical Performance Battery (SPPB) has been widely accepted as a tool to assess the risks related to mobility [14]. In accordance with the SPPB, we chose two functional tests to evaluate the ability to walk and lift the body upward. At the same time, we also chose a self-answering questionnaire because the ADL of each subject may be determined by the objective function of the motor system as well as the self-rated functional ability. Therefore, these three items are intended to represent different aspects of the motor function.

The Two-Step test was reported to have a good correlation with walking speed and also takes lesser time and space for measuring. Our results showed a wide range of values, indicating good sensitivity. Many of the functional tests used for the aged population are too easy for the younger population and have the problem of the ceiling effect [15]. The Two-Step test has the advantage of detecting high performance in the younger population as well as low performance in the elderly population. Therefore, we assume that this test is a good screening tool for evaluating horizontal mobility, i.e., walking ability. From the view point of safety, appropriate caution should be taken to prevent falling in applying Two-Step test to late elderly people ( ≥75) who already have dysfunctions in their locomotive organs. If the examiner recognizes any risk in performing this test, other measures should be considered.

The Stand-Up test which generates a non-parametric score is correlated to knee extension torque divided by body weight [10, 16]. Since the Chair-Stand test, a more detailed functional test for standing up, is also correlated to knee extension force [8], the Stand-Up test is expected to be correlated with the Chair-Stand test. This test is supposed to reflect vertical mobility, which is a different aspect than that reflected by the Two-Step test. Because the motion of this test is strongly associated with daily life, ordinary people can understand the meaning of this test and realize the weakness of their limb when they mark a lower score. Such characteristic of this test may contribute to the promotion of public awareness to motor function and may cover the disadvantage of this test, i.e. less sensitivity as a non-parametric measure with few grades.

The analysis of test–retest reliability revealed satisfactory reliability for both the Two-Step test (ICC >0.8) and Stand-Up test (Kappa value >0.7). In the scoring process of these functional tests, the decision whether the subject succeeded in the task is determined by the achievement of the final standing position for longer than 3 s without any additional steps. According to the criteria, we expect little discrepancy between observers, assuming good inter-observer reproducibility.

A self-answering GLFS questionnaire has several domains covering pain, physical function, basic ADL, instrumental ADL, and anxiety [7]. Because the concept of locomotive syndrome focuses on the quality of life of people with locomotive disorders, the assessment should involve both functional tests and subjective status.

From the view point of implementation of health-related screening systems, such programs should have the following characteristics. First, the test should be performed easily in limited space and should not take long time. Second, the parameters should have enough range so that there is no ceiling or floor effect when used in the target population. Finally, the interpretation of the tests should be relatively clear to the subjects, so that they understand the meaning of the results, and use the results to improve their exercise habit. The present study revealed that the three tests met all these requirements. Therefore, we propose the use of these three tests, named the “Short Test Battery for Locomotive Syndrome”, for screening the health of locomotive organs and for evaluating the need for therapeutic intervention [17, 18]. As for screening, we need to define the criteria to introduce the subjects to a secondary assessment. Further study of a larger number of subjects is needed to provide an accurate average score and the standard deviation, which would identify the subjects with motor function problems.

The present study had some limitations. The number of subjects was not large enough to define the cut-off average score and standard deviation of each age group. The aged subjects in the current study were recruited at medical check-ups. Because those who come to periodic medical check-ups tend to have high health awareness, there might be a selection-bias to choose elderly subjects who are in relatively better condition than general population. Further studies are required to enroll general population in defined regions. In addition, from the current cross-sectional data, we could not conclude about the predictability of these tests for future loss of independence in locomotion. The mean scores of healthy people would be used for a screening purpose and in an awareness program for health in locomotive organs. Regarding the prevention strategy for the high-risk group, who are older or have already been diagnosed with motor dysfunction, we need to determine clear criteria with cut-off values for each test. Such cut-off values would be used to categorize subjects into moderate- or high-risk groups for losing their independence in the near future. To define the cut-off value, we need to evaluate data from longitudinal studies which include these parameters.

In conclusion, we propose a screening test program which consists of two functional tests and one self-answering questionnaire to evaluate the health of locomotive organs in the adult population. Such standardized measuring system may facilitate conducting clinical trials and establishing solid evidence, and may promote public awareness about the importance of health on locomotive organs.
